# Autism Spectrum Disorder and Long-Term Survival in Attenuated Molybdenum Cofactor Deficiency Type A: A Case Report From Saudi Arabia

**DOI:** 10.7759/cureus.100887

**Published:** 2026-01-06

**Authors:** Abdulkarim O Alanazi, Nouf F Alshammari, Waleed Alsuhibani

**Affiliations:** 1 Department of Psychiatry, Prince Sultan Military Medical City, Riyadh, SAU; 2 Department of Psychiatry, King Abdulaziz Medical City, Riyadh, SAU

**Keywords:** attenuated phenotype, autism spectrum disorder, mocs1, molybdenum cofactor deficiency, neurodevelopmental disorders

## Abstract

We report the case of an 18-year-old male with genetically confirmed molybdenum cofactor deficiency type A (MoCD-A) due to a homozygous pathogenic MOCS1 variant. He presented in infancy with hypotonia and developmental delay and experienced a generalized tonic seizure at 20 months of age, followed by long-term seizure remission. His clinical course was notable for preserved motor function, below-average cognitive ability, marked speech delay, autism spectrum disorder (ASD), and prominent inattentive symptoms. Metabolic testing demonstrated elevated urinary xanthine, hypoxanthine, and S-sulfocysteine levels, supporting impaired sulfite oxidase activity. Neuroimaging revealed a small, focal hypodensity in the left basal ganglia without progressive changes.

This case illustrates an attenuated phenotype of MoCD-A, with survival into adulthood and detailed neuropsychiatric characterization, expanding the recognized clinical spectrum and underscoring the importance of considering metabolic etiologies in patients with overlapping developmental and behavioral features.

## Introduction

Molybdenum cofactor deficiency (MoCD) is an extremely rare, autosomal recessive, neurometabolic disorder resulting from impaired biosynthesis of the molybdenum cofactor, which is required for sulfite oxidase and other molybdenum-dependent enzymes [1]. Accumulation of sulfite and S-sulfocysteine leads to significant neurotoxicity and early neurologic deterioration in most affected infants [2]. MoCD is genetically classified into types A, B, and C, depending on pathogenic variants in MOCS1, MOCS2, MOCS3, or GPHN, with MoCD-A representing the most common subtype [1,3,4].

Classically, MoCD presents in the neonatal period with refractory seizures and encephalopathy, often resembling hypoxic-ischemic injury [1,5]. Natural-history data show high early mortality and profound developmental impairment, with characteristic biochemical features such as elevated S-sulfocysteine and low uric acid [5]. In recent years, however, reports have described attenuated or late-onset forms with fewer seizures, partial motor preservation, and more variable developmental outcomes [3,6]. Despite this emerging phenotype, psychiatric and neurobehavioral manifestations - particularly autism spectrum disorder (ASD) and higher-functioning developmental profiles - remain largely undocumented, due to the predominance of severe, early-fatal presentations.

The true prevalence of MoCD remains unknown, as available data are derived primarily from case reports and small series; current estimates suggest that only a limited number of cases have been documented worldwide, with incidence varying across regions and appearing higher in populations with increased consanguinity [2]. To date, regional reports have focused almost exclusively on severe neonatal MoCD, with no documented cases describing psychiatric or higher-functioning developmental outcomes in MoCD-A [5,7]. This highlights a gap in understanding the broader neuropsychiatric spectrum of MoCD survivors.

This study aims to present a rare case of MoCD-A with preserved motor function, seizure remission, and prominent neuropsychiatric features, including ASD and inattentive symptoms. The objective is to expand clinical awareness of atypical MoCD-A presentations and to underscore the need for metabolic evaluation in children with overlapping developmental and behavioral concerns.

## Case presentation

The patient is an 18-year-old male with a confirmed diagnosis of autosomal recessive MoCD-A (MOCS1-related), ASD, attention-deficit/hyperactivity disorder (ADHD), below-average cognitive ability, and a longstanding speech delay. ASD and ADHD (predominantly inattentive presentation) were diagnosed by specialist clinicians following longitudinal clinical assessments, with symptom profiles meeting the Diagnostic and Statistical Manual of Mental Disorders, Fifth Edition (DSM-5) diagnostic criteria [8]. He was born full-term via cesarean section following an uncomplicated IVF pregnancy, with no neonatal complications and immediate discharge with his mother. He is the third son of consanguineous parents (first cousins). His family history is notable for a sister with cerebral palsy, attributed to extreme prematurity, and a brother with a history of speech delay. Review of the family history across first- and second-degree relatives revealed no additional individuals with confirmed MoCD, related metabolic disorders, a history of seizures, or psychiatric illness. 

The mother first became concerned at around four to five months of age, when she noticed that the patient was hypotonic and delayed in achieving gross motor milestones. He first sat unassisted at seven months. At 20 months of age, he experienced a severe generalized tonic seizure, was admitted to the hospital, and was started on antiepileptic therapy. He remained on carbamazepine for approximately five years, after which he became seizure-free, with no recurrence for more than 12 years, until the present.

Motor development remained delayed throughout early childhood. He walked independently at the age of two years after receiving physiotherapy; however, a mild, unsteady gait persisted, with toe-walking noted intermittently in subsequent clinical assessments. Fine motor delay was also documented, with difficulty writing and forming letters. Speech development was significantly delayed: he began speaking around age 5, and by age 9, he had developed marked dysarthria and persistent expressive language delay. Social reciprocity was limited from early childhood, and he exhibited restricted interests, a preference for routine, and poor peer interaction. He was also followed by ophthalmology for bilateral congenital ptosis and significant astigmatism, with preserved extraocular movements and normal fundus examinations. These findings remained stable over time and did not require surgical intervention.

The patient’s neurodevelopmental assessment at nine years of age demonstrated a nonverbal IQ of 86, corresponding to the 18th percentile, consistent with below-average cognitive ability. Subtests showed significant scatter, with weaknesses in form completion and sequential processing. During evaluations, he was described as cooperative but inattentive, with poor eye contact and limited expressive language. The test was administered under standard clinical procedures, and no copyrighted materials or test items were reproduced; their use complied with the publisher’s terms.

Throughout childhood and early adolescence, he displayed persistent behavioral concerns, including irritability, demanding behavior, tantrums, and aggression toward family members when frustrated or when his routine was disrupted. He demonstrated difficulty sustaining attention, forgetfulness, and declining school performance. Repeated behavioral assessments, incorporating reports from both parents and teachers, consistently demonstrated prominent inattentive symptoms, in keeping with an ADHD inattentive presentation. Episodes of impulsivity fluctuated in severity, and his mother frequently reported daily anger outbursts, shouting, and hitting.

The patient attended various educational settings throughout his schooling, including private schools, special education classes, and, later, specialized training centers. Despite multiple placements, he showed poor academic progress and required ongoing support for daily living skills, though he remained partially independent in basic self-care. Teachers consistently described him as forgetful, easily distracted, and academically behind peers. His school performance did not improve despite stable behavior in class, and his mother frequently requested reassessment due to ongoing concerns.

His behavioral challenges intensified during adolescence, leading to multiple referrals to pediatric neurology, developmental pediatrics, autism services, and child and adolescent mental health clinics. He completed a multidisciplinary autism program consisting of speech therapy, psychology, psychiatry, and occupational therapy interventions. Family therapy was also initiated to support parenting strategies and reduce overprotection. His mother was highly engaged in treatment, attending all parent-training sessions.

Pharmacologic interventions were trialed over several years. Risperidone was prescribed intermittently from childhood through adolescence, with doses ranging from 0.5 mg to 1 mg twice daily. It consistently reduced aggression and tantrums without significant adverse effects. Episodes of worsened irritability typically occurred when the medication was stopped or reduced. Later trials included methylphenidate for inattentive ADHD symptoms, initially tolerated but providing limited benefit, and eventually discontinued on neurology’s advice. At age 16, due to recurrent irritability and physical aggression, aripiprazole 10 mg was initiated, with subsequent improvement. He also received vitamin B12 injections for a documented deficiency at age 17.

**Figure 1:**
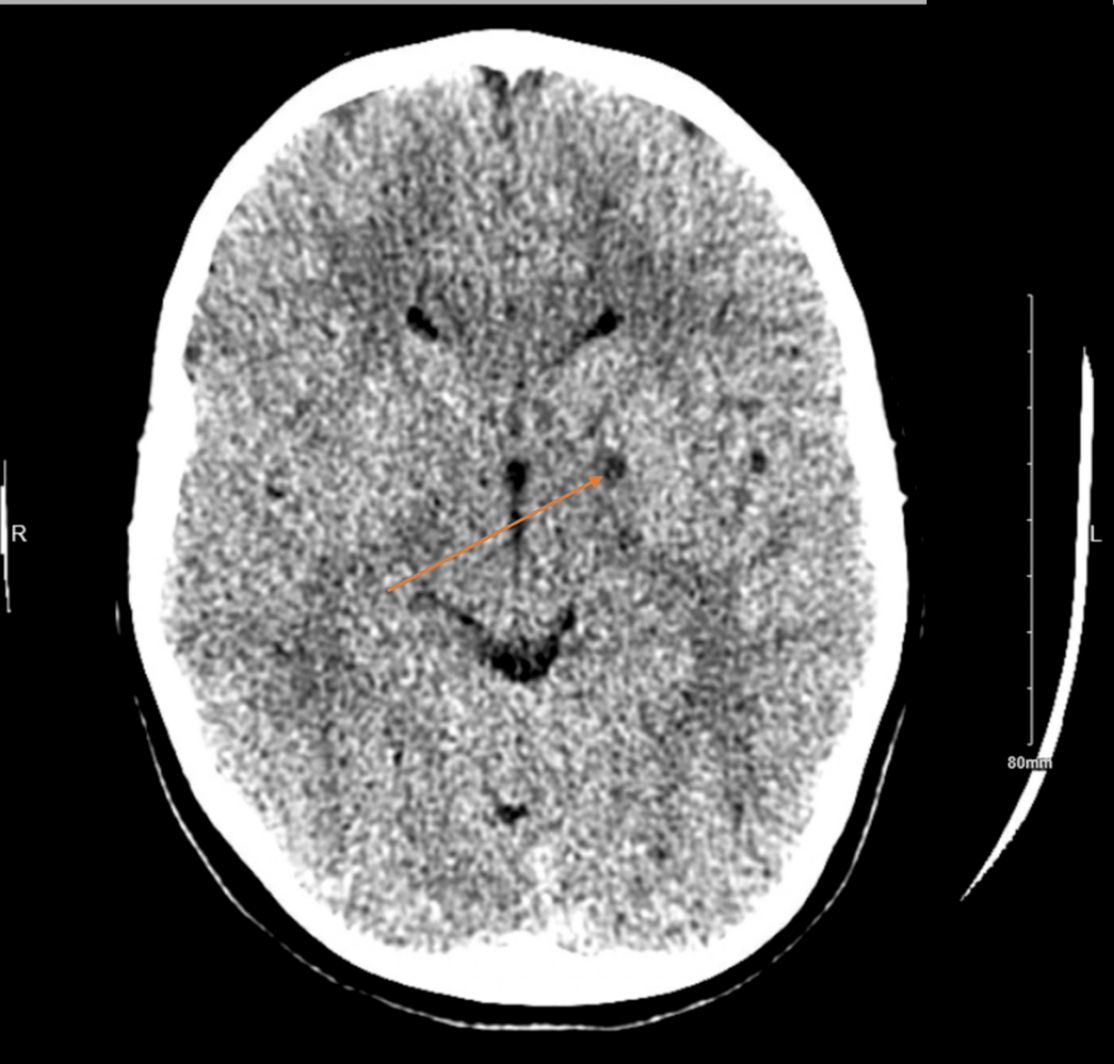
Non-contrast axial brain CT demonstrating a small, focal hypodensity within the left basal ganglia (arrow), measuring approximately 4 mm and reported as likely representing a prominent perivascular space, with no associated mass effect or hemorrhage.

A detailed genetic evaluation was performed following ongoing behavioral, cognitive, and developmental concerns. Whole-exome sequencing was performed on genomic DNA extracted from peripheral blood as part of routine clinical diagnostic testing, which identified a homozygous pathogenic variant in the MOCS1 gene (c.1102+1G>T), establishing the diagnosis of autosomal recessive MoCD-A. An incidental heterozygous pathogenic variant in CYP21A2 (cytochrome P450 family 21 subfamily A member 2), consistent with carrier status for congenital adrenal hyperplasia, was also detected. Targeted metabolic testing demonstrated elevated urinary xanthine, hypoxanthine, and S-sulfocysteine levels, supporting impaired sulfite oxidase activity and further confirming the diagnosis of MoCD-A. Additional investigations - including karyotype (46,XY), chromosomal microarray, very-long-chain fatty acids (VLCFA), plasma amino acids, urine organic acids, and acetylcholine receptor antibodies - were unremarkable. Neuroimaging included a non-contrast brain CT performed for evaluation of ataxia, which demonstrated a small, focal hypodensity within the left basal ganglia measuring approximately 4 mm, reported as likely representing a prominent perivascular space, with no associated mass effect, hemorrhage, or midline shift (Figure 1).

At present, the patient remains seizure-free, with persistent ASD features, speech delay, and below-average cognitive abilities. He continues to have intermittent episodes of irritability but has shown improved behavioral regulation with aripiprazole and structured routines. He is not currently employed and does not participate in formal academic education; instead, he attends a specialized developmental center daily, where he receives structured educational activities, speech therapy, behavioral interventions, and family support. Despite limitations in academic learning and social functioning, he maintains stable sleep, appetite, and general health. The family remains closely involved in his care and continues to seek supportive services to enhance his developmental progress.

## Discussion

MoCD classically presents in early infancy with rapidly progressive encephalopathy, intractable seizures, feeding difficulties, and severe neurologic decline [1,2]. The majority of affected neonates develop profound developmental delay, axial hypotonia with appendicular hypertonia, and characteristic neuroimaging abnormalities, including cortical atrophy and basal ganglia involvement [2,5]. Early mortality is common, with natural-history data showing that most infants with the early-onset form succumb within the first months to years of life, due to neurologic complications and secondary infections [2]. This classical presentation stands in contrast to the attenuated clinical course observed in our patient, who survived into adulthood with more preserved neurological functioning.

Recent literature has highlighted a broader clinical spectrum of MoCD, including late-onset and milder variants characterized by fewer seizures, partial preservation of motor skills, and extended survival [3,6]. These individuals often present beyond infancy with developmental delay, movement disorders, or episodic neurologic symptoms, rather than catastrophic neonatal encephalopathy [4]. Some case series have documented children who remain seizure-free for long periods, mirroring our patient’s 12-year seizure remission following early treatment [3,6]. Such findings reflect the increasing recognition that MoCD is not uniformly fatal in infancy, and that milder genotypes or residual enzymatic activity may contribute to prolonged survival.

Studies of MoCD survivors consistently report significant developmental delay; however, detailed cognitive profiling remains limited in published cohorts [2,3,9]. Children with attenuated forms typically exhibit delays in motor and language milestones, hypotonia, and varying degrees of intellectual disability [3,9]. Our patient’s below-average nonverbal IQ (86) and persistent speech delay are consistent with these patterns, yet the degree of functional independence and stable motor profile he achieved into adolescence is atypical compared with previously documented cases [2,3,9]. These findings emphasize the heterogeneity of cognitive outcomes and underline the need for long-term developmental follow-up in MoCD [2,9].

Although behavioral concerns, such as irritability or agitation, are occasionally mentioned in MoCD reports, formal psychiatric characterization is rarely documented, likely due to the early mortality of most affected neonates [2]. The literature contains only sparse descriptions of ASD-like features, attention deficits, or aggressive behavior in genetically confirmed MoCD cases [10,11]. Our patient demonstrated a fully characterized profile of ASD, ADHD - inattentive presentation - irritability, and episodic aggression, findings that have not been previously well described in the context of MoCD-A. This case, therefore, provides unique insight into the long-term neuropsychiatric phenotype of MoCD survivors [11].

The biochemical hallmark of MoCD - elevated sulfite and S-sulfocysteine - results in excitotoxic injury through overstimulation of N-methyl-D-aspartate (NMDA) receptors and impaired mitochondrial function [2]. This process preferentially affects highly metabolic brain regions, such as the basal ganglia, thalamus, and cerebral cortex, contributing to cognitive impairment and movement abnormalities [1,2]. In our patient, a small focal hypodensity within the left basal ganglia, identified on childhood CT imaging, may represent residual structural vulnerability from early metabolic injury, even in the absence of progressive radiologic changes. Such basal ganglia involvement may plausibly contribute to executive dysfunction, attentional difficulties, and emotional dysregulation, consistent with patterns described in other neurometabolic disorders [10,11]. Although ptosis is likely a coincidental finding, its presence raises the theoretical possibility of subtle involvement of eyelid motor pathways, given that mitochondrial dysfunction and excitotoxic injury can impact cranial nerve-related nuclei and periocular muscles in other metabolic disorders. The stability, symmetry, and lack of progression in this patient, together with normal extraocular movements and fundus examinations, strongly favor a benign congenital etiology. Nevertheless, documenting ptosis in a long-term MoCD-A survivor adds potentially informative phenotypic detail and may support future efforts to distinguish coincidental findings from underrecognized features in attenuated forms of the disease.

Consanguinity is a known risk factor for autosomal recessive metabolic disorders, and published reports from the Middle East describe clusters of MoCD among children from consanguineous families [3,7]. However, nearly all regional cases mirror the classic neonatal phenotype, with severe early neurologic decline and limited survival [7]. To our knowledge, no previously published Middle Eastern case has provided a detailed description of long-term neurodevelopmental, cognitive, and psychiatric outcomes in a genetically confirmed MoCD-A survivor. This highlights the rarity of our patient’s presentation and adds valuable regional data to the literature.​​​​​

Our patient’s early seizure history initially suggested an epileptic encephalopathy; however, long-term seizure freedom and relatively mild neurological impairment obscured the metabolic etiology. Diagnostic delays in attenuated MoCD have been reported, as atypical presentations often mimic more common neurodevelopmental disorders [12]. His overlapping ASD and ADHD symptoms, combined with progressive speech delay, further complicated the clinical picture and led to multiple referrals before genetic testing established the definitive diagnosis. This underscores the importance of considering inborn errors of metabolism in children with mixed developmental, behavioral, and neurologic symptoms [11,12].

While disease-modifying therapy (fosdenopterin) is now available for early-onset MoCD-A, its benefits are greatest when initiated in the neonatal period, and its use in older children remains unstudied [2]. In long-term survivors like our patient, management focuses on behavioral interventions, speech therapy, educational support, and psychiatric care [11]. Antipsychotic medications, such as risperidone and aripiprazole - as used in this case - have been effective in managing irritability and aggression in neurodevelopmental disorders and were well tolerated here [11]. Multidisciplinary care involving neurology, psychiatry, developmental pediatrics, and genetics remains essential for optimizing functional outcomes.

This case provides valuable longitudinal insight into an attenuated form of MoCD-A, supported by genetic confirmation and detailed developmental, cognitive, and psychiatric characterization. Although early biochemical data were unavailable - a common limitation in long-term survivors - our findings demonstrate that MoCD-A can manifest with higher-functioning trajectories, prolonged seizure remission, ASD, inattentive symptoms, and below-average cognitive ability. This expands the recognized phenotypic spectrum beyond the classical neonatal presentation and underscores the importance of considering metabolic etiologies in children with combined developmental and behavioral concerns, as well as the need for ongoing follow-up to better understand long-term neuropsychiatric outcomes.

## Conclusions

This case highlights an attenuated form of MoCD-A, characterized by long-term seizure remission, preserved motor abilities, ASD, and mild cognitive impairment - features that broaden the known phenotypic spectrum of this disorder. Definitive diagnosis was achieved through whole-exome sequencing, underscoring the critical role of genetic testing in identifying atypical and late-presenting metabolic disorders that may otherwise be misclassified as primary neurodevelopmental conditions. Interpretation of these findings is limited by the single-patient design and the absence of early biochemical data, which restricts generalizability. Clinicians should consider metabolic etiologies in children presenting with combined developmental, behavioral, and psychiatric symptoms.
